# Combined Interval Training and Post-exercise Nutrition in Type 2 Diabetes: A Randomized Control Trial

**DOI:** 10.3389/fphys.2017.00528

**Published:** 2017-07-25

**Authors:** Monique E. Francois, Cody Durrer, Kevin J. Pistawka, Frank A. Halperin, Courtney Chang, Jonathan P. Little

**Affiliations:** ^1^School of Health and Exercise Sciences, University of British Columbia Okanagan Kelowna, BC, Canada; ^2^Kelowna General Hospital, Kelowna Cardiology Associates Kelowna, BC, Canada

**Keywords:** dairy, exercise, lifestyle, body composition, glycemic control, endothelial function, blood pressure

## Abstract

**Background:** High-intensity interval training (HIIT) can improve several aspects of cardiometabolic health. Previous studies have suggested that adaptations to exercise training can be augmented with post-exercise milk or protein consumption, but whether this nutritional strategy can impact the cardiometabolic adaptations to HIIT in type 2 diabetes is unknown.

**Objective:** To determine if the addition of a post-exercise milk or protein beverage to a high-intensity interval training (HIIT) intervention improves cardiometabolic health in individuals with type 2 diabetes.

**Design:** In a proof-of-concept, double-blind clinical trial 53 adults with uncomplicated type 2 diabetes were randomized to one of three nutritional beverages (500 mL skim-milk, macronutrient control, or flavored water placebo) consumed after exercise (3 days/week) during a 12 week low-volume HIIT intervention. HIIT involved 10 X 1-min high-intensity intervals separated by 1-min low-intensity recovery periods. Two sessions per week were cardio-based (at ~90% of heart rate max) and one session involved resistance-based exercises (at RPE of 5–6; CR-10 scale) in the same interval pattern. Continuous glucose monitoring (CGM), glycosylated hemoglobin (HbA_1c_), body composition (dual-energy X-ray absorptiometry), cardiorespiratory fitness ($\dot{\text{V}}{\text{O}}_{2\text{peak}}$), blood pressure, and endothelial function (%FMD) were measured before and after the intervention.

**Results:** There were significant main effects of time (all *p* < 0.05) but no difference between groups (Interaction: all *p* > 0.71) for CGM 24-h mean glucose (−0.5 ± 1.1 mmol/L), HbA_1c_ (−0.2 ± 0.4%), percent body fat (−0.8 ± 1.6%), and lean mass (+1.1 ± 2.8 kg). Similarly, $\dot{\text{V}}{\text{O}}_{2\text{peak}}$ (+2.5 ± 1.6 mL/kg/min) and %FMD (+1.4 ± 1.9%) were increased, and mean arterial blood pressure reduced (−6 ± 7 mmHg), after 12 weeks of HIIT (all *p* < 0.01) with no difference between beverage groups (Interaction: all *p* > 0.11).

**Conclusion:** High-intensity interval training is a potent stimulus for improving several important metabolic and cardiovascular risk factors in type 2 diabetes. The benefits of HIIT are not augmented by the addition of post-exercise protein.

## Introduction

Worldwide more than 257 million people have type 2 diabetes, a figure projected to reach 395 million by 2030 (Shaw et al., 2010). Of those, 71% have hypertension and 40% have three or more coexisting chronic conditions, with cardiovascular disease the leading cause of mortality (Centers for Disease Control and Prevention, 2014). Accordingly, interventions that improve both glycemic control and reduce cardiovascular risk factors are central to reducing the burden of type 2 diabetes (Inzucchi et al., 2012). Lifestyle interventions, including exercise and nutrition are at the forefront for the prevention of diabetes complications (Inzucchi et al., 2012). Intensive glucose lowering with multiple pharmacological treatments leads to reduced microvascular complications (UK Prospective Diabetes Study Group, 1998), but the effect on macrovascular complications is unclear.

Large controlled trials and numerous experimental studies reveal the widespread benefits of exercise for people with type 2 diabetes (Marwick et al., 2009; Lin et al., 2015). The Look AHEAD (Action for Health in Diabetes) trial showed that moderate continuous exercise and a caloric restrictive diet leads to sustained reductions in cardiometabolic risk factors, diabetes complications, and health costs (Wing et al., 2013). However, the number of cardiovascular events between the intervention and control groups was not different. The addition of vigorous exercise may be required to elicit substantial changes in cardiovascular function (Baldi et al., 2016), as it appears that vigorous, but not low-moderate exercise, reduces cardiovascular disease (Tanasescu et al., 2002; Lee et al., 2003). Studies using higher exercise intensities, such as interval and resistance exercise, show strong effects on cardiometabolic outcomes (Wisløff et al., 2007; Weston et al., 2014).

Cardiorespiratory fitness is an independent predictor of all-cause mortality and cardiovascular events (Kodama et al., 2009). A recent meta-analysis revealed that the increase in fitness after interval training is ~2-fold greater than continuous training (Weston et al., 2014). In the longest trial to date comparing interval and continuous exercise in diabetes, Karstoft et al. (2013) randomized participants to 4 months interval walking (*n* = 12), energy and time-matched continuous walking (*n* = 12; 60-min, 5 days/week), or non-exercise control (*n* = 8). Greater improvements in fitness, body fat, and glycemic control were observed after interval compared to continuous walking and control (Karstoft et al., 2013). These findings clearly support the benefit of interval exercise, however the volume of exercise (300 min/week) is far greater than usually attained by the general population, many of whom cite lack of time as a considerable exercise barrier (Korkiakangas et al., 2009). Emerging evidence from small short-term trials show that low-volume high-intensity interval training (HIIT) rapidly improves glycemic control in type 2 diabetes (Little et al., 2011; Madsen et al., 2015). Low-volume HIIT involves alternating brief periods of vigorous exercise with periods of recovery, typically taking ~20 min per session and performed three times per week (Little et al., 2011). Further research is needed to confirm changes in cardiometabolic health outcomes after several months of low-volume HIIT in studies with larger sample sizes.

Sarcopenic obesity disproportionately affects people with type 2 diabetes (Park et al., 2009). Diminished lean muscle leads to poor physical functioning, glycemic control and cardiovascular health (Anton et al., 2013). The anabolic effects of exercise (Robinson et al., 2017) and high-quality protein (Reitelseder et al., 2011) are important for counteracting the age-associated decline in muscle, and when combined, provide synergistic effects on muscle protein synthesis (Esmarck et al., 2001; Hartman et al., 2007). In particular, it appears that consuming milk-protein after exercise promotes significant lean mass accretion and fat loss (Hartman et al., 2007; Josse et al., 2010). HIIT was recently shown to promote increased protein synthesis in the skeletal muscle of older adults, an effect linked to improved insulin sensitivity and mitochondrial function (Robinson et al., 2017). Combining HIIT with post-exercise protein supplementation therefore holds potential for maximizing skeletal muscle adaptations in order to improve cardiometabolic health outcomes, particularly in older adults.

The purpose of this study was to determine whether post-exercise milk augments the cardiometabolic benefits of low-volume HIIT in individuals with type 2 diabetes. The primary outcome of glycemic control was assessed across 3 days before and after the intervention using continuous glucose monitoring (CGM). Secondary outcomes of body composition, HbA_1c_, fasting blood parameters, fitness, blood pressure, and endothelial function were also examined to determine how low-volume HIIT impacted key cardiometabolic health parameters.

## Research design and methods

### Study design

This double-blind, randomized clinical trial was conducted at The University of British Columbia Okanagan between January 2015 and December 2016 (clinicaltrials.gov #NCT02251301). The Clinical Research Ethics Board (CREB #H14-01636) approved the study and participants provided written informed consent. Randomization was by a third-party using variable permuted block sizes with computer-generated random numbers and sealed envelopes. A researcher not involved in data analysis prepared the beverages so participants and study personnel were blinded to the beverage condition.

### Participants

Men and women between 40 and 75 years with physician-diagnosed type 2 diabetes (>6 months) were recruited from the Kelowna Diabetes Program via mail-out advertisements and sign-up sheets. Exogenous insulin users, diagnosed cardiovascular disease and diabetes complications, or contraindications to exercise (Thompson et al., 2013) were excluded. After telephone/email interviews interested participants attended a screening visit, which included a medical history questionnaire, physical activity readiness questionnaire-plus (PARQ+), and informed consent. Eligible participants then completed a 12-lead stress test using a modified Bruce protocol and a cardiologist provided clearance for vigorous exercise.

### Intervention

#### Experimental protocol overview

Fifty-three participants were randomized to one of three beverages; (i) low-fat milk, (ii) macronutrient control, or (iii) placebo, consumed after exercise (details below). Baseline and post-intervention outcomes were assessed over 5 days before and after the intervention (48–72 h after the last training session). Fasted blood and body composition measures were obtained on day 1 and CGM was performed across days 2–4 while participants followed a standardized diet. Blood pressure, endothelial function and fitness were assessed on day 5. Body weight, waist circumference, blood pressure, and endothelial function were also assessed at 6 weeks (Mid).

#### Exercise training

All groups performed supervised low-volume HIIT 3d/week for 12 weeks. To be consistent with exercise recommendations by the American Diabetes Association and the American College of Sports Medicine (Colberg et al., 2016) both resistance and cardio-based exercises were included in the HIIT program. The first and last sessions per week were cardio-based (cycle ergometer, treadmill, or elliptical) involving 1-min bursts of exercise at 85–90% of the participants' maximum heart rate (HR_max_; obtained during baseline $\dot{\text{V}}{\text{O}}_{2\text{peak}}$ test) with 1 min of easy recovery in between. The middle session each week involved whole-body resistance exercises (using resistance bands or multi-gym). Similar to cardio-based HIIT, each resistance exercise was performed for 1 min (as many repetitions as possible) at an intensity eliciting an RPE of 5 “hard” on the CR-10 scale (Borg, 1962) followed by 1 min of recovery. A 3-min warm-up and cool-down was performed with all sessions. The number of 1-min intervals in each session progressed from four in week one to ten in week six of training. Thereafter, 10 X 1-min intervals eliciting ~90% of HR_max_ (cardio-based) or RPE ~5 (resistance-based) were completed in each session. Previous short-term training studies in individuals with, and at risk for, diabetes, have shown this low-volume HIIT protocol is effective for improving cardiometabolic health (Little et al., 2011; Francois et al., 2016). A heart rate monitor was worn to closely prescribe intensity, and capillary blood glucose and blood pressure measures were obtained before and after each exercise session.

#### Post-exercise nutrition supplementation

After each session participants consumed 500 mL of either: (i) low-fat milk; (ii) milk protein macronutrient-matched control; or (iii) placebo (water), within 1 h. The beverages were designed to look and taste similar and distributed in opaque containers. To accomplish this, one-teaspoon of cocoa powder and ¼ teaspoon of stevia (Stevia In The Raw®, Cumberland Packing Corp; containing ~28 mg stevia) were added to each beverage. Low-fat milk was prepared from skim-milk powder (MedallionMilk Co., Canada) providing 187 calories, 19 g protein, 26 g carbohydrate, and <1 g of fat. Macronutrient-matched control (milk protein concentrate; Vitalus Nutrition Inc., Canada plus lactose; NOW® Foods, IL, US) provided 186 calories, 21 g protein, 24 g carbohydrate, and <1 g of fat; i.e., providing the same macronutrient and protein composition as milk but without the micronutrients and other bioactive factors. The placebo beverage provided <10 calories from the cocoa powder.

### Outcomes

#### Continuous glucose monitoring (CGM)

A continuous glucose monitor (iPro 2, Medtronic Inc.) was used to continuously measure blood glucose across 3 days before and after the intervention. CGM provides valuable insight (that a one-off fasting blood or HbA_1c_ sample cannot) into glycemic variability and the magnitude of postprandial excursions across several days under free-living conditions (Klonoff, 2005). The CGM continuously samples interstitial fluid from the abdomen, measuring glucose concentration every 5-min using the glucose oxidase reaction (Rossetti et al., 2010). Participants took capillary glucose samples (4X/d), which were used to retrospectively calculate retrospective blood glucose concentration via an algorithm within the online software program (CareLink Pro, Medtronic; Rossetti et al., 2010). All food, drink, and medication were recorded (including time eaten, amount, brand) for pre-testing, and then replicated exactly for post-intervention.

The primary outcome was 24-h average glucose (from 00:00 to 23:55), calculated as the mean of the 3 CGM days. Standard deviation of 24-h blood glucose and mean amplitude of glycemic excursions (MAGE; Molnar et al., 1970) were calculated from the same 24-h periods to assess glycemic variability.

#### Body composition

Waist circumference (WHO Expert Consultation, 2008), height and weight (Seca 700, Hamburg, Deutschland) were measured to the nearest 0.1 cm and 0.1 kg, respectively. Percent fat, visceral adipose tissue (VAT) and lean body mass (LBM) were measured by dual-energy X-ray absorptiometry (Hologic Discovery DXA, MA, USA). All measures were performed and analyzed by the same researcher, with calibrations and quality control testing performed daily.

#### Cardiorespiratory fitness ($\dot{\text{V}}{\text{O}}_{2\text{peak}}$)

$\dot{\text{V}}{\text{O}}_{2\text{peak}}$ was assessed using a maximal incremental ramp test on a cycle ergometer (Lode Excalibur, Netherlands) with continuous sampling of expired gases (Parvomedics TrueOne2400, USA). Beginning at 30 W, the test increased by 1 W every 4 s (15 W/min) until volitional exhaustion or contraindication (Fletcher et al., 2013). $\dot{\text{V}}{\text{O}}_{2\text{peak}}$ and RER were calculated from the highest 30-s average, while HR_max_ was recorded as the highest value obtained during the test.

#### Biochemical analyses

Fasting blood samples were collected by venipuncture into EDTA containing tubes, centrifuged for 15 min (1,550 g at 4°C) and the plasma stored at −80°C for subsequent batch analyses. Medications were withheld the morning of the fasting blood sample. Fasting glucose was measured by the hexokinase method, high-sensitivity C-reactive protein by latex particle enhanced immunoturbidimetric assay and triglycerides by the enzymatic glycerol kinase and glycerol phosphate oxidase method. Pre and post-intervention samples were analyzed concurrently in duplicate (average coefficient of variation 6.8%) on a clinical chemistry analyzer (Chemwell 2910, Awareness Technologies) using assays from Pointe Scientific (MI, USA). HbA_1*c*_ was analyzed from a separate EDTA tube by a medical laboratory that routinely performs this analysis according to the National Glycohemoglobin Standardization Program (NGSP).

#### Blood pressure and endothelial function

All measures were assessed 4 h postprandial, after abstaining from alcohol and caffeine for 12 h and, within participants, at the same time of day with meal and medication standardized. After 20 min of rest in a supine position, blood pressure was measured manually using the auscultatory method, at least twice to the nearest 2 mmHg.

##### Flow-mediated dilation

Brachial artery flow-mediated dilation (FMD) is an important prognostic indicator of endothelial function and incident cardiovascular disease (Yeboah et al., 2007). The ability of the vessel to dilate (%FMD) is measured in response to a physiological (shear stress) stimulus (Thijssen et al., 2011). In the current study, brachial artery FMD was assessed according to current guidelines (Thijssen et al., 2011). Briefly, simultaneous measures of diameter and blood velocity were obtained with high-resolution ultrasound (Terason 3200), 2 cm from the antecubital fossa. Data were collected over a 1-min baseline, for the last 30 s of a 5 min period of forearm ischemia (pneumatic cuff inflated 60 mmHg above systolic blood pressure) and for 3 min thereafter.

##### Brachial artery dilatory capacity

The peak blood flow and diameter response to ischemic handgrip exercise provides an index of resistance artery size or remodeling and the maximal dilatory capacity (Naylor et al., 2005). This is important since changes in artery function (%FMD) with exercise training are thought to occur rapidly (i.e., first few weeks) after which are superseded by changes in structure, potentially concealing further changes in function (Tinken et al., 2010). After 15 min of rest, following the FMD procedure, baseline diameter and blood velocity were recorded for 1 min. This was followed by 5 min of forearm ischemia (as above), including 3 min of isotonic handgrip exercise (1 contraction every 2 s using a dynamometer) between 1-min periods of ischemia alone (Naylor et al., 2005). Again recording resumed 30 s before cuff deflation and continued for 3 min thereafter.

Absolute FMD (peak diameter – baseline diameter), %FMD (peak – baseline diameter/baseline diameter), and time to peak diameter were measured using custom designed edge-detection and wall-tracking software, which minimizes user bias (Woodman et al., 2001). This protocol is routinely performed in our lab using the methods outlined in Francois et al. (2016); coefficients of variation for diameter and %FMD are 2.1 and 7.3%, respectively.

#### Quality of life (QoL)

Participants completed the Medical Outcomes Study Short Form 36 (SF-36) questionnaire before and after the intervention (McHorney et al., 1994). The SF-36 is a self-report QoL questionnaire; the scores are used to provide two norm-based T scores, physical component summary (PCS) and mental component summary (MCS).

### Diet and exercise standardization

Participants maintained their usual diet, lifestyle, and medication habits throughout the testing and training sessions, verified by physical activity and diet records. Baseline dairy consumption was assessed using a food frequency questionnaire, and dietary intake before and during the study was assessed using 3-day diet records analyzed using FoodWorks16 (The Nutrition Company, NJ, USA). Baseline activity was examined using both accelerometry (Actigraph GT3x, FL, USA) over a 7-day period to assess minutes of moderate-vigorous physical activity (MVPA, Freedson et al., 1998 cut-points) and a Godin leisure-time exercise questionnaire (Godin and Shephard, 1997; Table 1).

**Table 1:** Baseline characteristics of participants.

	**Milk (*n* = 18)**	**Macronutrient control (*n* = 16)**	**Placebo (*n* = 19)**
Sex	11 F	12 F	11 F
Age (y)	62 ± 8	56 ± 9	55 ± 9
BMI (kg/m^2^)	36 ± 7	35 ± 6	33 ± 6
Years of diagnosis	6 ± 6	7 ± 7	5 ± 6
**MEDICATIONS**			
Lifestyle only	5	5	3
Metformin	10	11	13
Sulfonylureas	6	1	3
SGLT2 inhibitors	1	2	3
DPP4 inhibitors	1	2	3
GLP1 analogs	1	2	0
Lipid lowering	9	7	7
Antihypertensive	7	6	8
**BASELINE PHYSICAL ACTIVITY**			
LTPA score	17 ± 15	14 ± 10	21 ± 17
MVPA (min/day)	14 ± 15	13 ± 13	30 ± 19
Dairy intake (servings/day)	2.3 ± 2.4	2.7 ± 2.1	2.1 ± 1.6

*F, Females; LTPA, Leisure-Time Physical Activity; MVPA, Moderate-Vigorous Physical Activity*.

### Statistical analyses

#### Sample size

Using means and standard deviations from previously published data on the change in CGM assessed hyperglycemia in type 2 diabetes after HIIT (Little et al., 2011), power calculations determined that *n* = 17 per group would be sufficient to detect a 30% reduction in glucose (Cohen *d* = 0.7) with a power of 80% and alpha of 0.05.

#### Statistics

Analyses were performed on all participants that completed the intervention. Characteristics of the intervention groups are shown in Table 1. Linear mixed models using SPSS 22.0 (SPSS, Chicago, Illinois) examined changes in trial outcomes (pre-post or pre-mid-post) between groups. Significant interactions were probed with pre-planned contrasts comparing the change within each group, whereas isolated significant main effects of time were examined by pairwise comparisons with groups collapsed using Least Significant Difference (LSD) test (Hopkins et al., 2009). Results are reported as means and standard deviations with 95% confidence limits. Magnitude based inferences were used to identify clinically meaningful changes in major outcomes using techniques described by Batterham and Hopkins (2006). The threshold for clinically beneficial changes in 24-h glucose and HbA_1*c*_ were reductions of 0.5 mmol/l and 0.7%, respectively, based on the reduced risk for diabetes complications (Mazzone, 2010). For cardiorespiratory fitness an increase of 1 metabolic equivalent (MET) was used for a 15% risk reduction in cardiovascular disease (Kodama et al., 2009). For %FMD +1% was used, based on the 13% risk reduction in cardiovascular events (Inaba et al., 2010). In line with previous studies, a 2 mmHg reduction in MAP was considered to be the smallest clinical threshold change for BP (Cook et al., 1995). The clinically meaningful difference in QOL was determined as a change >3 points (Warkentin et al., 2014).

## Results

### Participant compliance and adverse events

**Figure 1:**
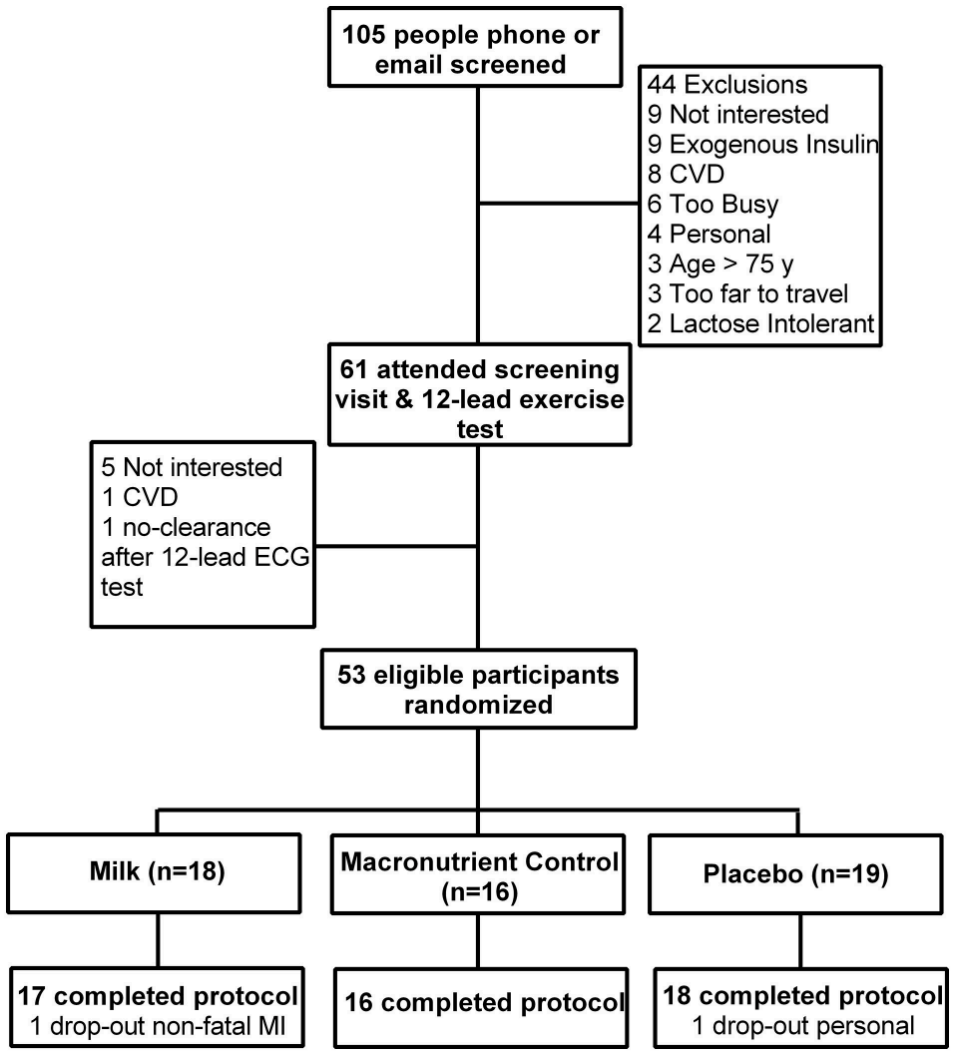
Consolidated Standards of Reporting Trials (CONSORT) flow diagram.

Figure 1 shows the CONSORT flow diagram of study progression. Fifty-three participants were eligible after screening; four required additional 24-h blood pressure monitoring (*n* = 2) and stress echo (*n* = 2) cardiologist clearance following the 12-lead ECG stress test. Baseline characteristics of randomized participants are shown in Table 1. The majority (51/53) were of European descent, while two were Southeast Asian (2/53).

Of the 53 participants randomized, 51 successfully completed 36 sessions of HIIT in 12 ± 1 wk. One participant suffered a non-fatal myocardial infarction in week eight (after 23 HIIT sessions) and one dropped out for personal reasons. There were no reports of hypoglycemia after exercise or at home throughout the intervention. Exercise sessions were rescheduled on 10 occasions (*n* = 6 due to sickness and *n* = 4 due to systolic blood pressure >144 mmHg prior to exercise). No musculoskeletal injuries were reported as a result of the training. $\dot{\text{V}}{\text{O}}_{2\text{peak}}$ testing was truncated in three participants because systolic pressure exceeded 250 mmHg during the test (Fletcher et al., 2013). For CGM analyses three participants were excluded due to sensor failure (*n* = 1) and medication changes (*n* = 2; required reduced medication). All other analyses are reported for *n* = 51 unless otherwise stated. Overall the exercise intensity achieved was 88 ± 7% of HR_max_ during cardio-based intervals, and an average RPE of 5 ± 1 and 4 ± 1, for cardio- and resistance-based intervals, respectively.

### Glycemic control

**Figure 2:**
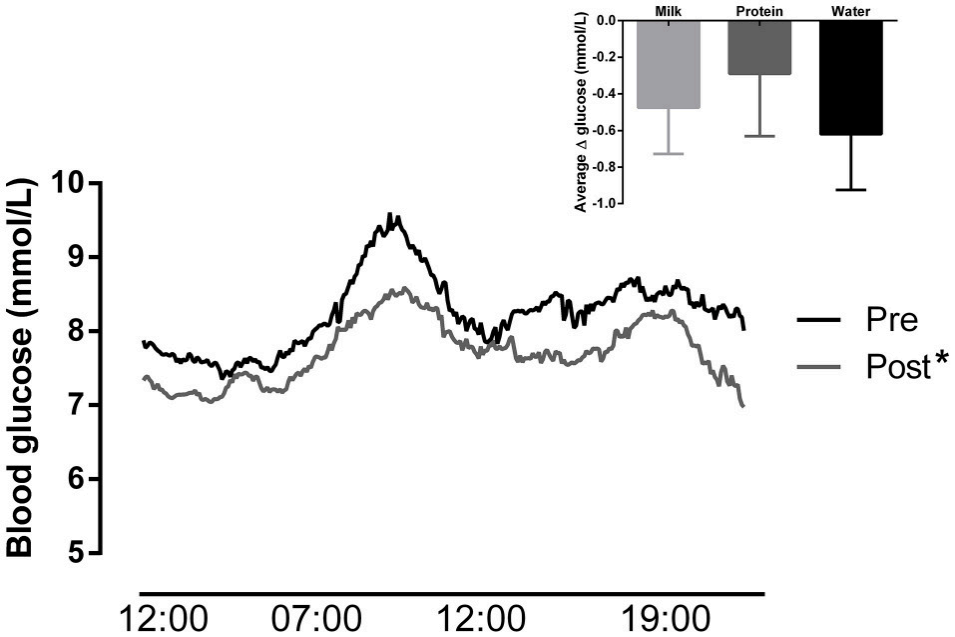
Continuous blood glucose across 24-h (*n* = 48) before and after the intervention (groups collapsed, ^*^main effect of time: *p* = 0.01). Inset: Change in blood glucose after the intervention in the milk, protein, and water groups.

There was a significant reduction in mean 24-h glucose following 12 weeks of HIIT (by −0.5 ± 1.1 mmol/L, Figure 2) with no difference between groups (Table 2). The probability that the change in glucose was clinically beneficial was 54% (95% CI: −0.8, 0.1 mmol/L). Glycemic variability assessed by both *SD* (by −0.33 ± 0.78 mmol/L) and MAGE (by −0.98 ± 2.27 mmol/L) was significantly reduced, with no differences between groups (Table 2). HbA_1c_ was significantly reduced after 12 weeks of HIIT (by −0.22 ± 0.39%, Figure 3) with no differences between groups (Table 2). The probability that the change in HbA_1c_ was clinically beneficial was 0% (95% CI: −0.33, 0.16%), with the change being most likely trivial. Fasting glucose was not significantly different after HIIT in all groups (Table 2).

**Table 2:** Body composition, cardiorespiratory fitness, blood pressure, flow-mediated dilation, triglycerides, C-reactive protein, and glycemic control measures before and after 12 weeks of HIIT and nutritional beverage.

	**Milk (*****n*** = **18)**		**Macronutrient control (*****n*** = **16)**		**Placebo (*****n*** = **19)**		***P*****-value**	
	**Pre**	**Post**	**Pre**	**Post**	**Pre**	**Post**	**Interaction**	**Time**
**BODY COMPOSITION**								
Mass (kg)	97.7 ± 19.3	96.8 ± 20.5	95.9 ± 17.3	94.5 ± 17.3	89.5 ± 21.1	89.1 ± 20.9	0.46	0.03^*^
VAT (g)	1057 ± 335	1033 ± 316	1007 ± 260	981 ± 212	815 ± 337	802 ± 285	0.75	0.25
**CARDIORESPIRATORY FITNESS**								
$\dot{\text{V}}{\text{O}}_{2\text{peak}}$ (L/min)	1.7 ± 0.4	2.0 ± 0.7	1.8 ± 0.5	2.0 ± 0.6	1.9 ± 0.5	2.1 ± 0.5	0.53	<0.01^*^
**BLOODS**								
HbA_1c_ (%; mmol/mol)	7.1 ± 0.8	6.9 ± 0.7	6.9 ± 0.8	7.0 ± 0.7	6.9 ± 0.8	6.6 ± 0.9	0.92	<0.01^*^
	54 ± 9	52 ± 8	54 ± 8	53 ± 8	51 ± 8	49 ± 9		
Fasting glucose (mmol/L)	8.6 ± 2.3	8.3 ± 1.7	9.2 ± 1.9	9.5 ± 2.3	8.9 ± 2.7	8.5 ± 2.1	0.35	0.53
Triglycerides (mg/dL)	149 ± 82	152 ± 70	161 ± 62	139 ± 65	152 ± 93	142 ± 80	0.36	0.17
C-reactive protein (mg/dL)	7.1 ± 10.3	4.4 ± 5.3	4.7 ± 4.3	4.9 ± 4.7	3.7 ± 4.1	3.1 ± 3.6	0.33	0.21
**CGM GLUCOSE CONCENTRATION**								
24-h mean (mmol/L)	8.4 ± 1.4	7.7 ± 1.2	8.1 ± 1.4	7.8 ± 1.7	8.4 ± 2.1	7.8 ± 1.5	0.74	0.01^*^
SD (mmol/L)	1.6 ± 1.0	1.3 ± 0.5	1.6 ± 0.6	1.1 ± 0.4	1.7 ± 0.8	1.5 ± 0.7	0.51	0.01^*^
MAGE (mmol/L)	4.3 ± 3.5	3.1 ± 1.3	4.1 ± 2.0	2.8 ± 1.3	4.1 ± 2.2	3.7 ± 1.6	0.60	0.02^*^
**BLOOD PRESSURE**								
Systolic (mmHg)	130 ± 10	119 ± 7	132 ± 13	129 ± 9	128 ± 13	117 ± 11	0.03^#^	<0.01^*^
Diastolic (mmHg)	79 ± 6	75 ± 5	83 ± 11	79 ± 6	81 ± 7	75 ± 7	0.20	<0.01^*^
**FLOW-MEDIATED DILATION**								
Absolute FMD (mm)	0.020 ± 0.01	0.027 ± 0.01	0.018 ± 0.01	0.024 ± 0.01	0.019 ± 0.01	0.023 ± 0.01	0.61	<0.01^*^
Baseline diameter (mm)	0.41 ± 0.10	0.41 ± 0.09	0.41 ± 0.08	0.41 ± 0.07	0.41 ± 0.07	0.42 ± 0.07	0.77	0.71
Time to peak (s)	64 ± 26	57 ± 25	60 ± 30	46 ± 23	56 ± 21	50 ± 21	0.75	0.05^*^
Total energy intake (Kcal/day)	2053 ± 881	2039 ± 898	1810 ± 525	2017 ± 706	1912 ± 629	1888 ± 710	0.35	0.25

*HbA_1c_, Glycosylated Hemoglobin; BMI, Body Mass Index; VAT, Visceral Adipose Tissue; MAP, Mean Arterial Pressure; FMD, Flow Mediated Dilation; TE, Total Energy*.

* *Time effect p < 0.05*.

# Interaction group^*^time p < 0.05.

**Figure 3:**
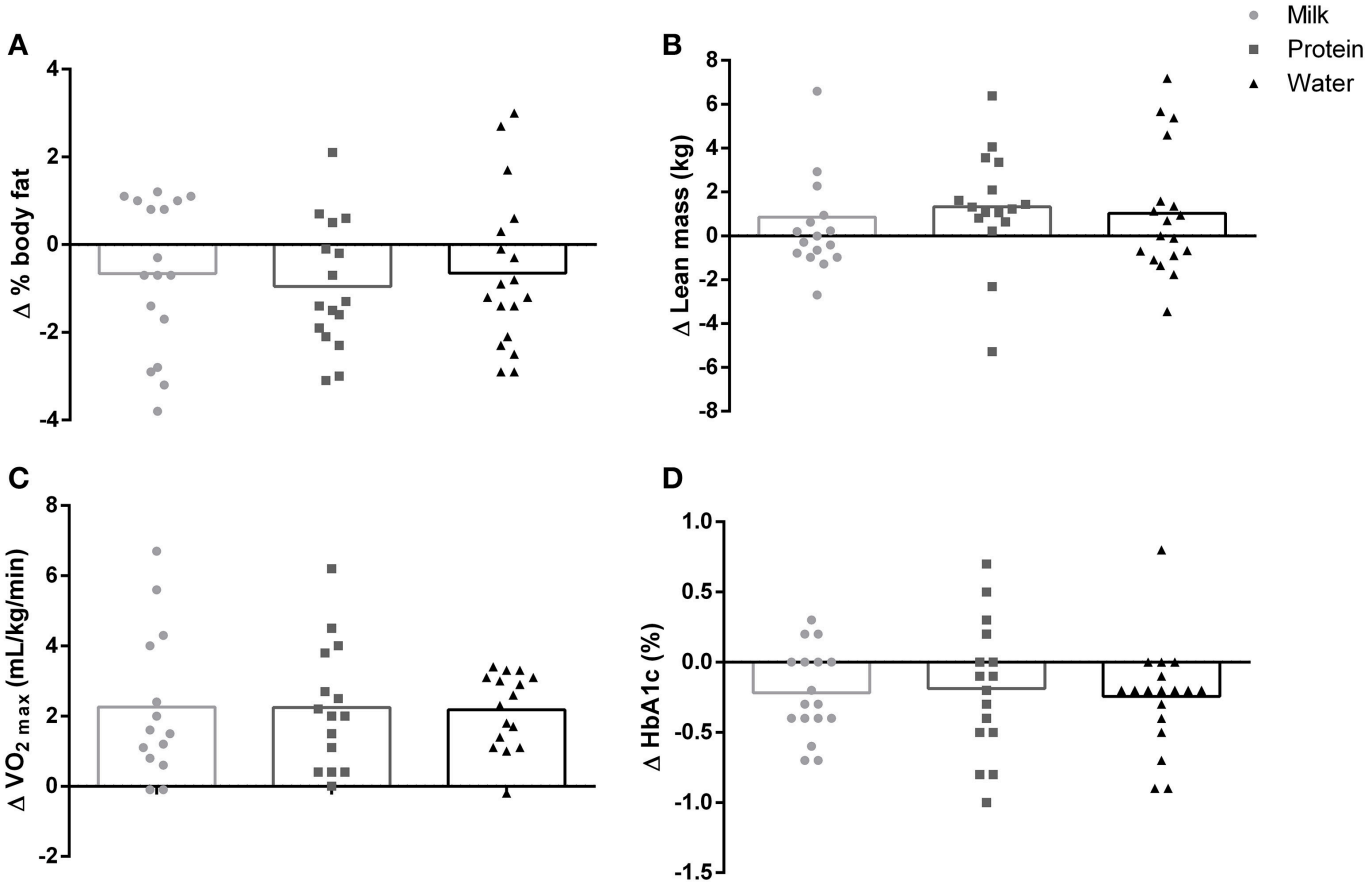
Change from pre intervention for **(A)** % body fat, **(B)** lean body mass, **(C)** cardiorespiratory fitness ($\dot{\text{V}}{\text{O}}_{2\text{peak}}$) and **(D)** glycosylated hemoglobin (HbA_1c_) in the milk, protein, and water groups (all main effect of time *p* < 0.05, no group interaction *p* > 0.05).

### Body composition

**Figure 4:**
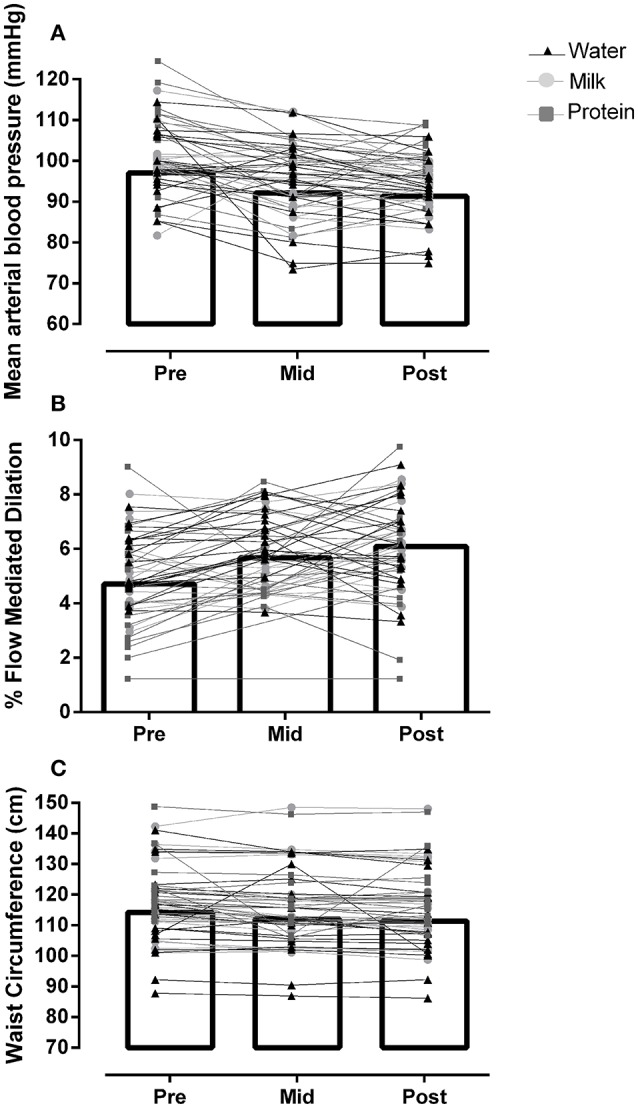
Data for **(A)** mean arterial blood pressure (MAP), **(B)** percentage flow-mediated dilation (%FMD), and **(C)** waist circumference, before (Pre), after 6 weeks (Mid), and 12 weeks (Post) mean for all participants (bar graph, all main effect of time *p* < 0.05) and individual data (line and symbols per beverage group, no group interaction *p* > 0.05).

Body mass was significantly lower after 12 weeks of HIIT (by −0.9 ± 3.9 kg, Table 2), with no difference between groups. There was a significant reduction in waist circumference after 12 weeks of HIIT (by −2.9 ± 3.5 cm, main effect of time: *p* < 0.01) with no difference between groups (Interaction: *p* = 0.21, Figure 4). Percent body fat was significantly reduced (by −0.76 ± 1.63%, main effect of time: *p* = 0.02) and lean body mass significantly increased (by +1.07 ± 2.76 kg, main effect of time: *p* = 0.01) after 12 weeks of HIIT, with no difference between groups (Interactions: all *p* > 0.83, Figure 3).

### Cardiorespiratory fitness ($\dot{\text{V}}{\text{O}}_{2\text{peak}}$) and blood pressure

$\dot{\text{V}}{\text{O}}_{2\text{peak}}$ significantly increased 9.8% after 12 weeks of HIIT (main effect of time: *p* < 0.01, Figure 3) with no difference between groups (Interaction: *p* = 0.55). The probability that the change in fitness was clinically beneficial was 5% (95% CI: 1.8, 3.1 mL/kg/min), with the change being 95% very likely trivial.

Mean arterial blood pressure was significantly reduced after 12 weeks of HIIT (by −5.7 ± 7.0 mmHg, main effect of time: *p* < 0.01) with no difference between groups (Interaction: *p* = 0.11, Figure 4). The probability that the change in MAP pre-post intervention was clinically beneficial was 99% (95% CI: −9, −2 mmHg).

### Flow-mediated dilation

%FMD significantly increased after 12 weeks of HIIT (by +1.4 ± 1.9%, main effect of time: *p* < 0.01), with no difference between groups (Interaction: *p* = 0.72, Figure 4). The probability that the change in %FMD was clinically beneficial was 94% likely (95% CI: 0.86, 1.94%). Absolute FMD also increased after HIIT (Table 2), with no difference between groups. Time to peak dilation was significantly lower (by 9.1 ± 31.1 s, Table 2) after 12 weeks of HIIT, with no difference between groups. Peak dilator capacity did not change across the intervention; Pre: 9.6 ± 5.2%, Mid: 8.1 ± 4.2%, Post: 10.4 ± 3.6% (main effect of time: *p* = 0.36).

### Quality of life

PCS scores significantly increased after 12 weeks of HIIT (*n* = 49, by 8.1 ± 12.1, main effect of time: *p* < 0.01) with no difference between groups (Interaction: *p* = 0.11). The probability that the change in PCS pre-post intervention was clinically beneficial was 99% likely (95% CI: 4.4, 11.8). The change in MCS post-intervention was different between groups (*n* = 49, Interaction: *p* = 0.02); *post hoc* testing revealed significant improvements in the protein group (+12.1 ± 9.69, *p* < 0.01) but not skim-milk (−1.1 ± 13.5, *p* = 0.79) or placebo (+5.6 ± 10.7, *p* = 0.06).

### Dietary intake records

Analysis of the 3-day diet records collected before and during the last week of the intervention showed no difference in the total daily energy intake between groups and/or across time (Table 2). Macronutrient composition of the diet was not different between groups (*p* = 0.32), or across time: for % carbohydrate (Pre: 48.0 ± 12.5% vs. Post: 48.4 ± 13.0% of total energy, *p* = 0.47), % protein (Pre: 20.4 ± 4.9% vs. Post: 19.9 ± 4.9% of total energy, *p* = 0.15) and % fat (Pre: 30.3 ± 12.5% vs. Post: 30.7 ± 13.3% of total energy, *p* = 0.49).

## Discussion

This study comprehensively examined the cardiometabolic benefits of HIIT in individuals with type 2 diabetes. We show for the first time that 12 weeks of low-volume HIIT, with or without post-exercise milk or protein, improves glycemic control, blood pressure, cardiorespiratory fitness, body composition, and endothelial function. Low-volume HIIT therefore appears to be a feasible and efficacious lifestyle intervention, involving minimal time and resource, to improve health in type 2 diabetes. Reducing the interval length and total exercise time has previously been shown to increase enjoyment and compliance (Martinez et al., 2015). To this end, we experienced very low dropout rates and high compliance to low-volume HIIT. In addition, we show that 12 weeks of HIIT improves quality of life, similar to previous studies in hypertensive (Molmen-Hansen et al., 2012) and heart failure (Wisløff et al., 2007) patients.

Exercise interventions generally result in modest weight loss, however exercise promotes lean mass accretion; which has important implications for whole-body metabolism, glucose disposal, and quality of life (Anton et al., 2013). Indeed, in the current study HIIT significantly increased lean mass and reduced body fat. Although weight loss was not a goal of the intervention, participants lost, on average, ~0.9 kg of body mass, which was a statistically significant change yet small in magnitude (~1%). Generally studies report significant benefits of weight loss in the magnitude of 5–7% (Wadden et al., 2012) but it is possible that improvements in some cardiometabollic outcomes were related to the small amount of weight loss seen. Consuming high-quality protein after exercise is known to further potentiate muscle protein synthesis (Esmarck et al., 2001; Hartman et al., 2007). Despite this, comparable changes in body composition and cardiometabolic health were seen with post-exercise milk, milk-protein, or water. In agreement, Parr et al. (2016) found changes in body composition after a combined resistance training and diet intervention were independent of the amount and type of protein (high/low dairy). Epidemiological data shows an inverse relationship between low-fat dairy consumption and the risk of type 2 diabetes (Aune et al., 2013) and the addition of four servings of low fat dairy per day has been shown to improve insulin resistance (Rideout et al., 2013). Therefore, additional milk/protein supplementation (e.g., on non-exercise days) may have been needed to elucidate effects of nutritional supplementation. Indeed, some previous studies showing benefits on lean mass have provided milk/protein after exercise 5 days per week (Hartman et al., 2007; Josse et al., 2010). However, ~20 g of post-exercise protein (similar to the current study) has been shown to maximize muscle protein synthesis (Churchward-Venne et al., 2016). To this end, a non-exercising control group may be required to detect effects of post-exercise protein added to a potent training intervention such as, HIIT. However, we feel a non-exercise control group in type 2 diabetes is unethical since numerous studies have shown worsening of glycemic control and cardiovascular risk factors in control group participants (Church et al., 2010; Karstoft et al., 2013).

Current research suggests that HIIT is more effective than continuous training for improving insulin resistance (Jelleyman et al., 2015). A recent meta-analysis revealed that absolute changes in HbA_1c_ are 0.5 and 0.25% greater with HIIT than control and continuous exercise, respectively (Jelleyman et al., 2015). The small, yet significant change in HbA_1c_ in the current study is in line with previous HIIT interventions (Madsen et al., 2015; Cassidy et al., 2016) yet robust changes in 24-h glucose were observed (Figure 2). Interestingly, the changes in 24-h glucose are similar to Karstoft et al. (2013) after 4 months of high-volume HIIT (300 min/week). This is an important finding given the perceived time barrier to exercise participation in type 2 diabetes (Korkiakangas et al., 2009). The use of CGM is a strength as it allows for additional insight into the changes in postprandial hyperglycemia and overall glycemic variability (Klonoff, 2005). Mean 24-h glucose and glycemic variability were reduced by 7 and 23%, respectively, after HIIT, regardless of post-exercise nutritional supplementation. Glycemic variability may be a stronger predictor than HbA_1c_ for diabetes complications (Praet et al., 2006). Previous research also shows that HIIT has the potential to improve beta cell function as Madsen et al. (2015) demonstrated an increase in the oral disposition index and HOMA-%β after 8 weeks. The mechanisms underlying the improvements in glycemic control could not be ascertained from the present study design but likely involve a combination of improvements in peripheral insulin sensitivity, beta cell function, and hepatic insulin resistance (Karstoft et al., 2014; Madsen et al., 2015; Cassidy et al., 2016). Collectively, these findings show the potential of HIIT to improve several underlying aspects of glycemic dysfunction in type 2 diabetes.

The added benefits of vigorous exercise for cardiovascular health are well known (Marwick et al., 2009; Baldi et al., 2016) and many studies have demonstrated superior cardiovascular effects of HIIT compared to continuous training (Wisløff et al., 2007; Marwick et al., 2009; Weston et al., 2014). Extending on this work, we observed an ~10% increase in cardiorespiratory fitness, a 6 mmHg reduction in MAP and ~1.4% improvement in FMD following 12 weeks of HIIT in type 2 diabetes. In itself, cardiorespiratory fitness is a strong predictor for cardiovascular mortality with each MET increase associated with a 10–20% improvement in survival (Kodama et al., 2009). Although only a 0.7 MET increase was observed, this is in line with previous low-volume HIIT studies (Madsen et al., 2015) and participants are likely to have gained significant health benefits given their low baseline fitness (<6 MET). A meta-analysis showed that the greatest mortality benefits occur for even small increases in fitness for those progressing from the least fit category (Kodama et al., 2009). Furthermore, the low-volume nature of the HIIT protocol involved only 45–78 min of exercise per week with one session being resistance training. The combination of resistance and cardio exercise may be superior to either type alone for improving health in type 2 diabetes (Church et al., 2010). Indeed, in hypertensive patients blood pressure is reduced more with combination training than cardio alone (Lamberti et al., 2016); the 5–6 mmHg reduction is in line with the current study. Our findings suggest that HIIT performed as combined aerobic and resistance exercise clearly promotes beneficial cardiovascular adaptations in type 2 diabetes patients.

In conclusion, we show that low-volume HIIT, with or without post-exercise milk or protein supplementation, improves metabolic and cardiovascular risk factors in individuals with type 2 diabetes. The combination of resistance and aerobic-based HIIT increases lean mass, reduces fat mass, and improves endothelial function. This study, the largest and longest low-volume HIIT study in type 2 diabetes to date, provides further evidence that HIIT is a feasible and efficacious exercise intervention to improve glycemic control, cardiovascular fitness, and body composition.

## Author contributions

MF, JL, and CD designed the study. MF, CD, KP, FH, and CC conducted the research. MF, JL, KP, and FH analyzed the data. MF and JL wrote the initial draft of the manuscript. All authors edited the manuscript and approved the final draft.

### Conflict of interest statement

The authors declare that the research was conducted in the absence of any commercial or financial relationships that could be construed as a potential conflict of interest.

## Abbreviations

HIIT: High-intensity interval training
FMD: Flow mediated dilation
$\dot{\text{V}}{\text{O}}_{2\text{peak}}$: Cardiorespiratory fitness
CGM: Continuous glucose monitoring
MAGE: mean amplitude of glycemic excursions
QoL: Quality of Life
HR_max_: Peak heart rate
RPE: Rating of perceived exertion
VAT: Visceral Adipose Tissue.
